# A Benchmark Data Set to Evaluate the Illumination Robustness of Image Processing Algorithms for Object Segmentation and Classification

**DOI:** 10.1371/journal.pone.0131098

**Published:** 2015-07-20

**Authors:** Arif ul Maula Khan, Ralf Mikut, Markus Reischl

**Affiliations:** Institute for Applied Computer Science, Image and Data Analysis Group, Karlsruhe Institute of Technology, Karlsruhe, Baden-Wuerttemberg, Germany; Jiangnan University, CHINA

## Abstract

Developers of image processing routines rely on benchmark data sets to give qualitative comparisons of new image analysis algorithms and pipelines. Such data sets need to include artifacts in order to occlude and distort the required information to be extracted from an image. Robustness, the quality of an algorithm related to the amount of distortion is often important. However, using available benchmark data sets an evaluation of illumination robustness is difficult or even not possible due to missing ground truth data about object margins and classes and missing information about the distortion. We present a new framework for robustness evaluation. The key aspect is an image benchmark containing 9 object classes and the required ground truth for segmentation and classification. Varying levels of shading and background noise are integrated to distort the data set. To quantify the illumination robustness, we provide measures for image quality, segmentation and classification success and robustness. We set a high value on giving users easy access to the new benchmark, therefore, all routines are provided within a software package, but can as well easily be replaced to emphasize other aspects.

## Introduction

Image processing is a means for automatically extracting image contents, often used in science (e.g. biological readouts (mouse [1], fish [2], insect [3]), surveying and mapping [4], particle accelerator [5] and man-machine interaction [6, 7]) or industry (nutrition industry [8, 9], quality supervision [10] or pick-and-place applications [11, 12], an overview of image processing in industry is given in [13].).

In computer vision applications, image processing routines need to be developed for image data sets containing a set of similar images. This happens in many real-time acquisition systems (surveillance camera etc.), lab equipment (high-throughput microscopic imaging etc.) or offline analysis of big databases containing similar images (objects in satellite images, human blood cells analysis in laboratory etc.).

In general, an image processing routine consists of the elements preprocessing and filtering, segmentation, interpretation and quantification, each consisting of further sub-units or operators building a so-called pipeline. The segmentation seeks to assign each pixel a property (e.g. being part of a structure or not), the interpretation assigns pixels with same properties to similar objects and the quantification assigns each object a feature vector of numbers describing the properties of each object. In some cases, classification algorithms are applied to assign a label to all found objects based on the object feature vector.

Each step of the pipeline contains parameters which are tuned by the developer. The outcome of the segmentation is highly dependent on the correct use of both, sub-routines and parameters. It depends, therefore, on the experience of the developer. Correctly parametrized pipelines deliver good results on the image data set they were designed for, however they often fail on unknown data.

To develop and evaluate new algorithms in comparison with standard methods, routines and parameter sets are validated using benchmark data sets with a clear ground truth about objects. Therefore, numerous benchmark data sets exist (e.g. data sets used in [14] for benchmarking and validation in biological image segmentation, [15] for cell image analysis by using simulated cell populations, [16] for semantic automatic image annotations using complex scenes, [17] for event recognition in surveillance videos and many others). Images in certain data sets contain highly complex objects or background as in large data sets e.g. [18–20] such as animals and vehicles against a complex background. Benchmark databases for traffic signs [21], a street scene with pedestrians, a lion sitting on grass, visual event recognition in videos etc. also exist. In other cases, there are sets with incomplete ground truth http://www.broadinstitute.org/bbbc/ such as images containing cells with only the total number of cells as ground truth without any information of the type and shape of the objects to be segmented.

Major problems in image processing are alterations in image data sets due to effects like shading, noise etc. Since different images deliver different results, algorithms are hard to compare. Therefore, not only the absolute quality of a segmentation algorithm for validation data sets is important, but also its ability to cope with unknown distortions, called robustness. To evaluate robustness, the segmentation quality in relation to the intensity of the alteration is a good measure. To derive this measure for a new routine, the benchmark must not only contain the ground truth for segments but also image variations of aforementioned effects. Furthermore, the strength of these effects needs to be given for each sample. Benchmarks known in literature do not deliver this data. Therefore, it is not possible to evaluate new image processing algorithms for robustness.

Furthermore, if the subsequent outcome of a classifier is to be evaluated not only a ground truth in segmentation but also in object labels is required. For classifier validation, there are plenty of benchmark data sets (IRIS [22] or WINE [23]). Benchmarks allowing image segmentation and object classification in combination with a quantified distortion have not yet been published. As well, there are robustness and evaluation measures for image segmentation [24], but no consistent methodology to evaluate both, the combination of image segmentation and classification.

This article introduces a new benchmark data set for the validation of robustness in image processing and classification. It contains easy-to-find segments of different object types (having different shapes, sizes, mean intensity values etc.) with given ground truth in segmentation and classification. Distortions like shading, noise and overlapping objects are inserted in varying intensities. Altogether, we introduce four data sets, each containing labeled segments of 5–9 object classes. The data sets contain a series of images with increasing shading intensity and noise level.

The distortion is quantified by calculating a fuzzy artifact level depending on amount of shading and intensity of noise. A measure for algorithm quality and outcome is provided by three measures, quantifying segmentation quality, segmentation overlap and classification accuracy.

To evaluate new image processing routines efficiently, we introduce an exemplary image processing pipeline and show how to apply the new framework. We prove the functionality comparing two basic image processing routines and deliver initial results on the data set regarding accuracy and robustness. The sources for the image processing routine, the robustness measures and the data set as well are freely available and downloadable https://sourceforge.net/projects/gait-cad/files/Benchmarks/hardware_items/.

## Materials and Methods

### Benchmark data set—irregular shaped solid hardware items

Details about data sets and information about object types are given in Table A in S1 Text. In order to evaluate the robustness, the image benchmark data set itself requires a quantification of image quality called artifact level and quality measures for segmentation and object classification.

**Fig 1:**
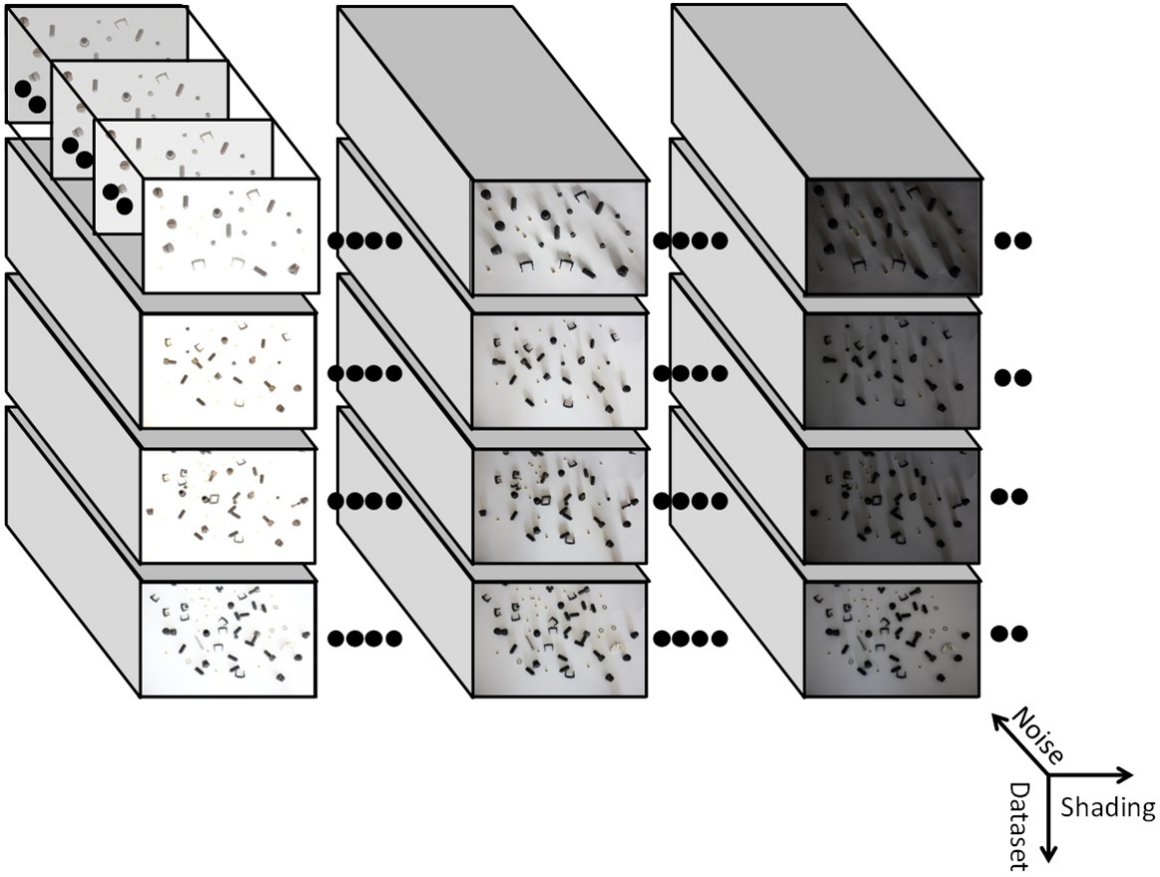
Benchmark data sets. Overview of images in *R* = 4 scenes corresponding to increasing artifact and noise levels

The benchmark consists of images **X** = ((*x*_*ij*_)) ∈ ℕ^*m* × *n*^ with *m* = 1000, *n* = 1500, *x*_*ij*_ ∈ [0, 255] with variations in brightness/shading and noise. There are *R* = 4 data sets, each combining *B* = 13 grades of shading and *N* = 14 grades of noise. Thus, altogether *B*⋅*R*⋅*N* = 728 images are contained in the data set (see Fig 1). Images are saved as 8bit TIFF-files, the naming convention is ‘benchdata_r_b_n.tif’, where *r*, *b*, *n* are data set number, brightness and noise level respectively. Each number is represented as two digits (e.g. ‘benchdata_02_01_03.tif’).

Shadowing and shading were introduced by two light sources from above and aside. Images were taken using a professional camera with a remote trigger in order to keep object positions same for *b* = 1, …, *B* lighting conditions (*b* = 1: very bright, no shading; *b* = 13: dark, lot of shading).

For each acquired image, artificial background noise in varying intensities was added. Altogether, 13 additional noise levels were generated using Gaussian-distributed random numbers with varying standard deviations. Thus, the image **X**(*r*, *b*, *n*) denoting data set *r*, brightness level *b* and noise level *n* is given by:

$$\begin{array}{c} x_{ij}\left(r,b,n\right)=f\left(x_{ij}\left(r,b,n\right)+x_{\text{rnd},ij}\right), \end{array}$$ (1)

where *x*_rnd,*ij*_ is a realization of the Gaussian-distributed random variable $X_{\text{rnd},ij}∼N(0,\sigma_{n}^{2})$ with $\sigma_{n}^{2}=125(n-1)$. The function *f* restricts possible values to [0, 255]:

$$\begin{array}{c} f\left(x\right)=\{\begin{array}{ccc} 0 & \;\text{if}\; & x<0\\ 255 & \;\text{if}\; & x>255.\\ x & \;\text{else} \end{array} \end{array}$$ (2)

**Fig 2:**
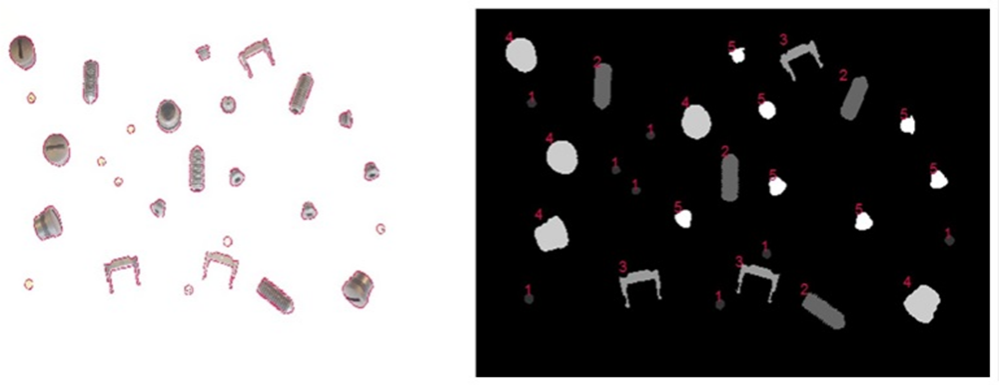
Representation of ground truth objects using color shading and numbers as labels. Left: Brightfield image **X** of a benchmark data set with marked object edges, right: Ground truth image **X**_truth_, with object types in numbers. Gray scales denote the value of *x*_*ij*,*truth*_ (0: black (background), 5: white)

The maximum number of objects in any data set is denoted as *O* and total class types are denoted as *K*. Objects are not aligned and multiple viewing directions are possible.For each image, a ground truth in object classification and segmentation is given: **X**_truth_ = ((*x*_*ij*_))_truth_ ∈ ℕ^*m* × *n*^ with *x*_*ij*, truth_ ∈ {0, …, *K*} (0: background, 1, …, *K*: objects). An exemplary image is depicted in Fig 2.

The ground truth is provided using full lighting conditions and semi-automatic labeling (first using Otsu segmentation [25] and then manual correction in Windows Paint Application), both in terms of their type and object boundaries (see Fig 2). See S2 Text for more details on manual labeling.

### Measures for image quality, segmentation and robustness

To evaluate the quality of a new image processing routine, on the one hand, image quality needs to be quantified, on the other hand segmentation and classification success needs to be measured. In this section, we briefly describe the motivation of all measures. The use of the given measures provides direct applicability for other users but is not mandatory and can also be replaced by tailor-made or more general pipelines. Therefore, all routines and evaluation measures are given in source code that can be downloaded and even manipulated.

To enable algorithm developers to incorporate robustness into their algorithms, we provided benchmark images with distortions and artifacts in varying strengths. The amount of distortion is measured by an artifact function *A*(*r*, *b*, *n*) which aggregates the shading level and the noise level. Shading is quantified using the mean pixel value in an image, noise is quantified by the parameter *σ*_*n*_ of the Gaussian-distributed noise. A monotonic fuzzification function *μ* ∈ [0, 1] based on the quantifying parameter delivers a mean to normalize the effects of shading and noise. *μ* contains tunable higher and lower bounds *α* and *β*, to suppress upper and lower outliers as shown in Fig A of S3 Text. Detailed descriptions are given in S3 Text.

To evaluate the outcome of an image processing routine, we use a quality criterion *Q*(*r*, *b*, *n*) based on the number of detected objects (Segmentation measure 1), their respective areas (Segmentation measure 2) and the classification accuracy based on the number of misclassified objects (Classification measure). It is specifically designed to incorporate further evaluation measures in addition to the necessary criteria for segmentation evaluation. Segmentation measure 1 compares the detected objects to the objects given in the ground truth and thus quantifies the outcome of object recognition. Segmentation measure 2 evaluates the pixel positions of the detected objects related to the positions of ground truth objects. It judges the quality of the foreground/background discrimination. The Classification measure is based on the difference between assigned classes by the classifier in comparison to true object classes. The total quality measure *Q*(*r*, *b*, *n*) fuzzifies and combines the three evaluation measures. The S4 Text contains all procedures in detail.

Using the aforementioned measures we derive a quality measure for robustness *R*. If the quality measure *Q*(*r*, *b*, *n*) is plotted over the artifact level *A*(*r*, *b*, *n*), algorithm 1 is called to be more robust, if its data-points exceed the data-points of algorithm 2 in their values. This is usually quantified using the area under the curve or by adapting a tansig-regression and evaluating the position of the inflection point.

To have an objective set of varying artifact levels for performance evaluation, we select images constituting of an image series **X**(*r*, *k*, *k* + 1) where *k* = 1, …, 13. An algorithm is then assigned the robustness measure

$$\begin{array}{c} R=\frac{\sum_{b=1}^{B}Q\left(r,b,b+1\right)}{B}. \end{array}$$ (3)

### Exemplary processing pipeline

**Fig 3:**
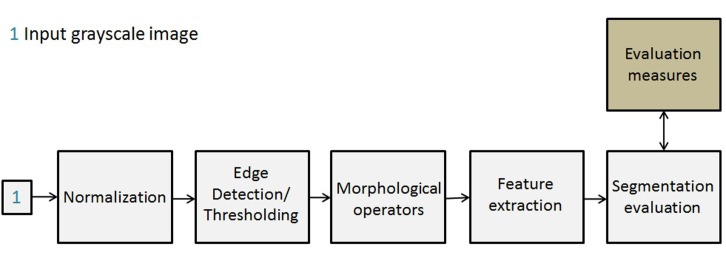
Exemplary pipeline for the segmentation of benchmark images.

We implemented an exemplary pipeline for standard algorithms given in Fig 3 to apply the aforementioned methods and thus to compare two or more subroutines in an image processing pipeline. This pipeline was used for testing standard algorithms by evaluation their performance on the benchmark data sets.

In this pipeline, a grayscale image is first normalized using 2% and 98% quantiles. The normalized image is then segmented using standard image segmentation techniques. Here, we want to use the benchmark to compare a standard Otsu operator to a Sobel edge detector [26]. The resulting binary image is then passed through morphological operations such as image hole filling, image opening and image border clearing in order to get reasonable objects. Each object is described by 8 features to design a Bayes classifier. Features are: area, mean intensity, maximum intensity, minimum intensity, solidity, median intensity, standard deviation of intensity values and eccentricity. Eccentricity is defined as the ratio of the distance between the ellipse foci and its major axis length. It is a scalar value between 0 and 1 where 0 is a circle and 1 is a line segment. This pipeline has been implemented as macro in the Gait-CAD software ([27]) developed in MATLAB and is provided with the implementation code.

## Results

We use the image processing pipeline given in Fig 3 to compare the segmentation steps i.e. Otsu operator versus a Sobel edge detector. As a native edge detection does not directly seek for objects, we expect the edge detector to deliver worse results. However, the subsequent hole filling delivers objects in undistorted cases. The robustness of each pipeline is quantified using Eq (3) and Data Set *r* = 1. This scene *r* = 1 was selected due to the lower number of foreground objects and less complex cases to make this demonstration more understandable.

**Fig 4:**
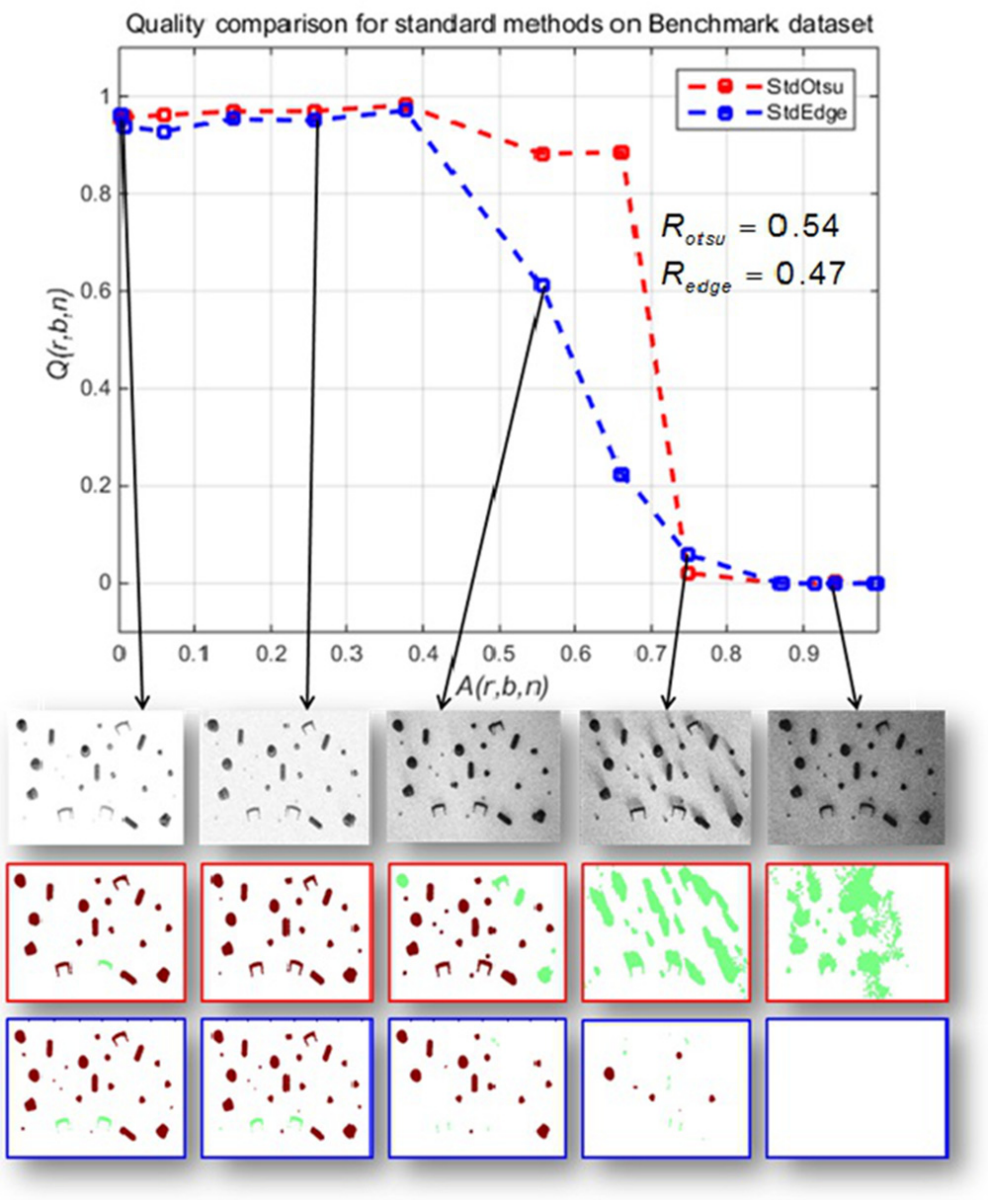
Results of benchmark data set *r* = 1. Total quality criterion *Q*(*r*, *b*, *n*) vs. increasing artifact level *A*(*r*, *b*, *n*) for image series with stepwise addition of both shading and noise for each successive image. The first row of images indicates original images from data set *r* = 1. The second row shows corresponding segmentation and classification results using Otsu’s method. The third row shows corresponding segmentation and classification results using edge detection method. Brown color represents correct classification of the segmented BLOB w.r.t corresponding ground truth BLOB and light green color shows an erroneous classification object. Robustness values for Otsu thresholding and Sobel edge detection are *R*_*otsu*_ = 0.54 and *R*_*edge*_ = 0.47 respectively.

The performance of the two pipelines, one using the Otsu operator and the other using the Sobel edge detector with respect to increasing artifact levels are given in Fig 4. The segmentation evaluation measures are integrated in total quality measure *Q*(*r*, *b*, *n*).

Fig 4 shows a decreasing performance of the segmentation algorithms indicated by *Q*(*r*, *b*, *n*) with increasing artifact levels (*A*(*r*, *b*, *n*)). To quantify the performance degradation, the robustness measures *R*_*otsu*_ and *R*_*edge*_ for Otsu thresholding and Sobel edge detection, respectively are given. *R* is given on a scale between 0 and 1 where larger values show high robustness. Both segmentation methods are found to be comparatively closer to each other. At higher artifact levels, edge detection fails to find the adequate number of objects as compared to Otsu thresholding but classification results are seen to be better than those obtained with Otsu segmentation. Wrong classification assignments occur if the artifact level is increased.

## Discussion

The presented framework contains images of the same scenes under varying illumination conditions and noise levels as well as the ground truth for segment detection and object type classification. Furthermore, we provide measures to evaluate artifact level, segmentation and classification quality. Thus, robustness evaluation of image processing and classification algorithms becomes possible and enables developers to compare image processing algorithms with respect to robustness. To this end, the development of algorithms with a focus on robustness in distorted data is accelerated. Parameters and structures can easily be evaluated and optimized.

Furthermore, not only the robustness of an image-processing pipeline can be evaluated but also segmentation quality. Parameters can be optimized with respect to given data quality. For example, if the quality of images is known to be bad, algorithms do not need to provide optimal results on good quality images.

In addition, the outcome of an image processing pipeline can be fed back to optimize its parameters. Therefore, users have a means to tune parameters in a pipeline not only to fit a special set of images but to be applied to a more general class of problems.

Basically each part of the framework (images, artifact level calculation, quality calculation, robustness) can individually be replaced, depending on the preferences of the developer. Even the images may be replaced by arbitrary images as long as the calculation of the artifact level (depending on the parameter to be used, here: mean pixel value) delivers reasonable results.

We provided all measures and routines in source code to allow other developers a uniform processing to compare their algorithms to standard algorithms.

Using the proposed benchmark, we have shown the effect of increasing artifact levels on the image segmentation outcome using standard algorithms. The proposed quality measures may be used for other object classification benchmark without a special robustness focus as well.

In our ongoing work, we use the presented framework to parametrize and evaluate new feedback-based image processing routines.

## Supporting Information

S1 Text Data and object information. **Table A**. Distribution of total object number *O* and maximum class type *O* in different scenes.
(PDF)

S2 Text Data Labeling. (PDF)

S3 Text Artifact level. **Fig A**. Spline based fuzzy artifact function.
(PDF)

S4 Text Quality measures for segmentation and classification. (PDF)
