# Supplementary material for: A Benchmark Data Set to Evaluate the Illumination Robustness of Image Processing Algorithms for Object Segmentation and Classification
Source: PLoS One. 2015 Jul 20;10(7):e0131098. doi: 10.1371/journal.pone.0131098 (PMC4508044; doi:10.1371/journal.pone.0131098)
Supplement: S1 Text — Table A. Distribution of total object number O and maximum class type O in different scenes. (PDF) [file pone.0131098.s001.pdf]

S1 Text

Data and object information. This data set is based on mostly fasteners as objects. In different scenes, different types of fasteners such as screws, bolts, nuts, washers, plastic hole plugs, plastic caps and clips are used. The objects in different scenes are distributed randomly. The number of objects and class types involved in each scene are given in Table A. Objects size do not vary greatly in individual scenes. However, in $r = 1$ objects are placed a bit closer to camera, therefore, bigger object support size can be seen when compared to the remaining scenes. The minimum and maximum support size is also given in Table A. Moreover, for $k = 8, 9$, only one object is used in order to introduce rare object in a scene.

Table A. Distribution of total object number O and maximum class type O in different scenes. It also includes minimum and maximum object support sizes in pixels for each scene r.

k	1	2	3	4	5	6	7	8	9
**Description of objects**	hex nut	set screw	plastic clip	plastic cap	plastic hole plug	hex head bolt	washer	wing nut	slotted head bolt
$r = 1$ **No. of objects**	8	4	3	5	7	0	0	0	0
$r = 1$ **Min. object size (pixels)**	628	6164	4896	6787	1660				
$r = 1$ **Max. object size (pixels)**	824	7072	5171	8076	2257				
$r = 2$ **No. of objects**	10	5	5	5	4	3	0	0	0
$r = 2$ **Min. object size (pixels)**	163	2369	1551	2805	598	4352			
$r = 2$ **Max. object size (pixels)**	253	2939	1751	3583	874	4558			
$r = 3$ **No. of objects**	21	8	8	9	4	2	0	0	0
$r = 3$ **Min. object size (pixels)**	69	2283	1351	2832	663	4023			
$r = 3$ **Max. object size (pixels)**	241	2953	1808	3458	808	4468			
$r = 4$ **No. of objects**	20	11	6	8	6	2	3	1	1
$r = 4$ **Min. object size (pixels)**	139	2198	1480	2678	590	4284	1116	6125	2327
$r = 4$ **Max. object size (pixels)**	244	3492	1792	3468	925	4599	1420	6125	2327
