# Supplementary material for: A Benchmark Data Set to Evaluate the Illumination Robustness of Image Processing Algorithms for Object Segmentation and Classification
Source: PLoS One. 2015 Jul 20;10(7):e0131098. doi: 10.1371/journal.pone.0131098 (PMC4508044; doi:10.1371/journal.pone.0131098)
Supplement: S2 Text — (PDF) [file pone.0131098.s002.pdf]

S2 Text

Data Labeling. This manual segmentation was made by only one observer. Images are of high resolution i.e. 1000×1500, a small pixel error in labeling does not cause segmentation methods and proposed evaluation criteria to deviate noticeably, neither are the object features greatly affected by small pixel error if any. However, in applications where very high accuracy of object delineation in terms of ground truth is required, more observers can be included to cross-check all images against provided ground truth.
