# Supplementary material for: A Benchmark Data Set to Evaluate the Illumination Robustness of Image Processing Algorithms for Object Segmentation and Classification
Source: PLoS One. 2015 Jul 20;10(7):e0131098. doi: 10.1371/journal.pone.0131098 (PMC4508044; doi:10.1371/journal.pone.0131098)
Supplement: S3 Text — Fig A. Spline based fuzzy artifact function. (PDF) [file pone.0131098.s003.pdf]

S3 Text

Artifact level. Artifact levels are introduced in data sets in order to give image processing algorithm developers a possibility to incorporate robustness in their algorithms. To quantify an algorithm's robustness versus shading and noise, we introduce an artifact level $A(r, b, n) \in [0, 1]$ which aggregates the shading level and the noise level.

The quantification of shading and noise is done using a fuzzification function $\mu \in [0, 1]$ based on a quantifying parameter θ (e.g. mean brightness or deviation). The overall artifact level *A(r,b,n)* is then calculated as:

$$A(r, b, n) = 1 - (1 - \mu_1)(1 - \mu_2). \tag{1}$$

Where, μ_1 and μ_2 are artifact functions as μ is monotonic with tunable higher and lower bounds α and β (see Fig A), to suppress a tunable percentage of higher and lower outliers which should not change the function value.

μ is defined as:

$$\mu(\theta; s, \alpha, \beta) = \begin{cases} 1, & \theta < \alpha \\ 1 - 2^{s-1}(\frac{\theta-\alpha}{\beta-\alpha})^s, & \alpha \leqslant \theta \leqslant \frac{\alpha+\beta}{2} \\ 2^{s-1}(\frac{\theta-\beta}{\beta-\alpha})^s, & \frac{\alpha+\beta}{2} < \theta \leqslant \beta \\ 0, & \theta > \beta \end{cases} \tag{2}$$

The parameter s defines the curvature: $s = 1$ is a trapezoidal function, $1 < s < \infty$ is a spline-based function, $s \to \infty$ delivers a step function.

To quantify the amount of shading and background present in $\mathbf{X}(r, b, n)$, we use the mean pixel value x_m across the whole image as a parameter $\theta = x_m$, since the benchmark contains dark objects in front of a bright background, higher x_m values mean less shading and vice versa. Furthermore, we set $s = 2, \alpha = 0$ and $\beta = 255$. Thus,

$$\mu_1 = \mu(x_m; 2, 0, 255). \tag{3}$$

On the other hand, the Gaussian variance is given as $\sigma_b^2 = (N - 1) \cdot 125$. Thus, increasing values of σ_b denote a higher level of artifacts and we introduce

$$\mu_2 = 1 - \mu(\sigma_b; 2, 10, 45). \tag{4}$$

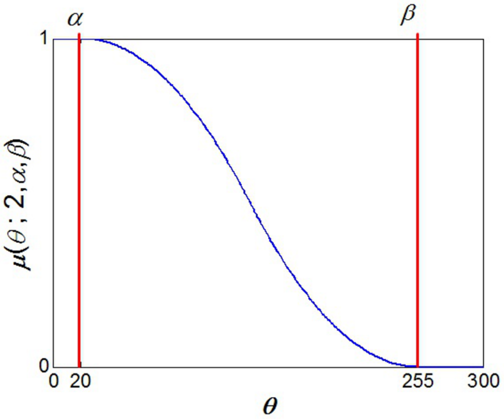

Fig A. Spline based fuzzy artifact function. The effect of parameter selection on spline based fuzzy function is shown i.e. $\alpha = 20$ and $\beta = 255$. These values does not correspond to the actual values used for μ but shown only for elucidating the effect of parameter selection.
