# Supplementary material for: A Benchmark Data Set to Evaluate the Illumination Robustness of Image Processing Algorithms for Object Segmentation and Classification
Source: PLoS One. 2015 Jul 20;10(7):e0131098. doi: 10.1371/journal.pone.0131098 (PMC4508044; doi:10.1371/journal.pone.0131098)
Supplement: S4 Text — (PDF) [file pone.0131098.s004.pdf]

S4 Text

Quality measures for segmentation and classification. Standard image segmentation algorithms such as Otsu thresholding, edge detection, clustering etc. are useful for segmenting identifiable objects against a static non-complex background. Since the benchmark contains the ground truth, supervised evaluation methods can be used and the quality of each image segmentation routine can be estimated. The supervised segmentation evaluation measures are based on the degree of similarity between image segmentation resulting from an application of a particular algorithm against a manually-segmented reference image. A variety of such measures exist and are mostly based on the number of pixels that are misclassified when compared to the image object pixels in the manually labeled image while penalizing pixels inversely proportional to distance from correct ones [1, 2]. Many methods use the difference in the number of objects detected with the ground truth ([3, 4]). Other methods based on object features extraction [5], edge-based image segmentation evaluation [6], using Pareto front [7] also exist.

Measures like Rand Index (RI), Jaccard Index (JI), Normalized Sum of Distances (NSD) and Hausdorff Metric (HM) are useful when evaluating 2D image segmentation [8, 9]. In RI based on true positive and negative image pixels, the image must contain useful information in its negative pixels, too. In our case, we need not our background to be segmented since we have foreground objects as ground truth against a background that contains no useful information. Therefore, we need to develop a criterion based on positive pixel values. In such cases, RI tends to deliver high values even if no foreground object is segmented because background constitutes most of the pixels in images of our data set. Moreover, false detections are to be penalized in such a criterion.

Here, we opted for fuzzy evaluation criteria since they are intuitively understandable. Our evaluation criteria are defined upon a segmented and classified image $\mathbf{X}_{\text{seg}} \in \mathbb{N}^{m \times n}$ with $x_{\text{seg},ij} \in \{0, ..., K\}$ (0: no segment, *1*,..., K: class label) and the given ground truth image $\mathbf{X}_{\text{truth}} \in \mathbb{N}^{m \times n}$ with $x_{\text{truth},ij} \in \{0, ..., K\}$. $\delta_{\text{seg},ij}$ and $\delta_{\text{truth},ij}$ represent binary pixel values of $\mathbf{X}_{\text{seg}}$ and $\mathbf{X}_{\text{truth}}$ respectively.

We introduce a quality criterion evaluating the number of detected objects (Segmentation measure 1) and their respective areas (Segmentation measure 2) and the classification accuracy (Classification measure) based on the number of misclassified objects. It is specifically designed to incorporate further evaluation measures in addition to the necessary criteria for segmentation evaluation.

1. Segmentation measure 1 (q_1): Difference in total number of objects detected in comparison to the ground truth.

   Let S be the set of all the BLOBs (Binary Large Objects) found in $\mathbf{X}_{\text{seg}}$ such that, $S = \{S_u | u = 1, ..., U\}$ and U is the total number of BLOBs found in $\mathbf{X}_{\text{seg}}$. S_u is a set of C_u pixel positions/coordinates and is defined as: $S_u = \{\{S_{ux,1}, S_{uy,1}\}, \cdots, \{S_{ux,C_u}, S_{uy,C_u}\}\}$.

   Similarly, let T be the set of all the BLOBs present in $\mathbf{X}_{\text{truth}}$ such that, $T = \{T_v | v = 1, ..., V\}$ and V is the total number of BLOBs present in $\mathbf{X}_{\text{truth}}$ and is given. Here, T_v is defined as $T_v = \{\{T_{vx,1}, T_{vy,1}\}, \cdots, \{T_{vx,C_v}, T_{vy,C_v}\}\}$ where, C_v denotes the number of pixels in BLOB T_v.

For each BLOB in S_u we determine the overlap with all ground truth BLOBs T_v sequentially. If the maximum overlap exceeds 1/3 (set heuristically) of T_v, we assume the BLOB S_u being present in the ground truth as well and add it to the set of correct segmented object S_c and delete the corresponding overlapping ground truth element from the complete set of V ground truth elements such that for a new S_u , $z = 1, \ldots , V - n_v$ (number of T_v BLOBs affected by overlap) and z is new number of remaining T_v elements. The formula for S_c in each iteration is given as:

$$S_c = \{S_u | \max(\text{card}(S_u \cap T_1), \ldots, \text{card}(S_u \cap T_Z)) > \frac{1}{3} C_u\}. \quad (1)$$

Where, Z indicates maximum number of elements left in z after overlap. Therefore, the criterion is described as:

$$q_1 = \frac{|\text{card}(T) - \text{card}(S_c)|}{\text{card}(T)} \quad (2)$$

The *a priori* knowledge for this measure is handled using fuzzy membership functions in order to describe it in the range of 0 - 1 according to (5) in **S3 Text**, such that:

$$\mu_1 = 1 - \mu(q_1; 2, 0, 1). \quad (3)$$

From the logical point of view, such a criterion engulfs all the cases of true positive (TF), true negative (TN), false positive (FP) and false negative (FN) detections. Since negative pixels are not the part of information we require, this criterion is be able to handle all possible case of positive pixels such as:

- one S_u overlaps one T_v i.e. TP (counted according to (2)).
- one S_u overlaps no T_v but just the background i.e. FP. Such cases are not counted as overlap according to (2).
- one S_u overlaps two T_v BLOBs. As a result of overlap, both BLOBs of T_v be deleted in new set T_z to be checked for next iteration, so it will not increase the overall count. In this way, (2) would not include the match twice but only once.
- two S_u BLOBs against one T_v. So, for the first S_u, the overlap will delete the corresponding T_v and only this count will be taken into account. In next iteration for the second S_u, no corresponding BLOB will be present in T_z.

Note: only the matches of BLOBs present in S are checked against BLOBs of T for the total count obtained to be checked against the total count of ground truth elements, which would again lead to just one match and yield a lower value of criterion.

2. Segmentation measure 2 (q_2): Non-overlapping pixels of the detected objects with the pixels of ground truth objects.

   It is described as:

$$q_2 = \frac{\sum_{i,j} |\text{sign}(\delta_{\text{truth},ij} - \delta_{\text{seg},ij})|}{\sum_{i,j} \text{sign}(\delta_{\text{truth},ij} + \delta_{\text{seg},ij})}. \quad (4)$$

   The *a priori* knowledge for this measure is handled using fuzzy membership functions in order to keep it consistent with other criteria here introduced here. It is here defined according to (5) in **S3 Text** as:

$$\mu_2 = 1 - \mu(q_2; 2, 0, 1). \quad (5)$$

3. Classification measure (q_3):

   For each image $\mathbf{X}_{\text{seg}}$, BLOBS are compared for class types using $\mathbf{X}_{\text{truth}}$. As BLOBS do not perfectly match the reference, the class assignment is needed to be synthesized. For each BLOB S_u in $\mathbf{X}_{\text{seg}}$, a classifier can assign a class based on a feature set f and then it is compared to class type K_v of the corresponding BLOB T_v in $\mathbf{X}_{\text{truth}}$.

   Let K_u represent the class assignment for each S_u and $\hat{K}_u$ represent the class assignment by a classifier (given by x_{seg}). Then

$$K_u = \operatorname*{argmax}_{k=0,\cdots,K} (\text{card}\left\{i \in \{1, \cdots, C_u\} | \text{x}_{\text{truth},S_{ux,i},S_{uy,i}} = k\right\}) \tag{6}$$

   such that, the quality criterion could be written as:

$$q_3 = \frac{1}{U} \sum_{u=1}^{U} |\text{sign}(K_u - \hat{K}_u)| \tag{7}$$

   This criterion (7) was then accordingly converted to a fuzzy criterion using:

$$\mu_3 = \mu(q_3; 2, 0, 1). \tag{8}$$

Using these criteria, the overall segmentation quality measure $Q_{\text{seg}}(r, b, n)$ for each image is then given as:

$$Q_{\text{seg}}(r, b, n) = \mu_1 \cdot \mu_2 \tag{9}$$

and the total quality measure is given as:

$$Q(r, b, n) = \mu_1 \cdot \mu_2 \cdot \mu_3. \tag{10}$$

References

1. Yasnoff WA, Mui JK, Bacus JW (1977) Error measures for scene segmentation. Pattern Recognition 9: 217–231.
2. Lee SU, Yoon Chung S, Park RH (1990) A comparative performance study of several global thresholding techniques for segmentation. Computer Vision, Graphics, and Image Processing 52: 171–190.
3. Yasnoff WA, Bacus J (1984) Scene-segmentation algorithm development using error measures. Analytical and Quantitative Cytology 6: 45–58.
4. Mezaris V, Kompatsiaris I, Strintzis M (2003) Still image objective segmentation evaluation using ground truth. In: Fifth COST 276 Workshop on Information and Knowledge Management for Integrated Media Communication. pp. 9–14.
5. Zhang Y, Gerbrands J (1994) Objective and quantitative segmentation evaluation and comparison. Signal Processing 39: 43–54.
6. Shin MC, Goldgof DB, Bowyer KW (2001) Comparison of edge detector performance through use in an object recognition task. Computer Vision and Image Understanding 84: 160–178.
7. Everingham M, Muller H, Thomas B (2002) Evaluating image segmentation algorithms using the pareto front. In: Computer Vision ECCV 2002, Springer. pp. 34–48.

8. Stegmaier J, Otte JC, Kobitski A, Bartschat A, Garcia A, Mikut R, et al. (2014) Fast segmentation of stained nuclei in terabyte-scale, time resolved 3D microscopy image stacks. PLoS ONE 9: e90036.

9. Coelho L, Shariff A, Murphy R (2009) Nuclear segmentation in microscope cell images: A hand-segmented dataset and comparison of algorithms. In: IEEE International Symposium on Biomedical Imaging: From Nano to Macro. IEEE, pp. 518–521.
